# Ultrasound-Assisted Extraction Optimization of α-Glucosidase Inhibitors from *Ceratophyllum demersum* L. and Identification of Phytochemical Profiling by HPLC-QTOF-MS/MS

**DOI:** 10.3390/molecules25194507

**Published:** 2020-10-01

**Authors:** Zhen Li, Zongcai Tu, Hui Wang, Lu Zhang

**Affiliations:** 1 State Key Laboratory of Food Science and Technology, Nanchang University, Nanchang 330047, China; 402337518013@email.ncu.edu.cn (Z.L.); wanghui00072@aliyun.com (H.W.); 2 National R&D Center for Freshwater Fish Processing, and Engineering Research Center of Freshwater Fish High-value Utilization of Jiangxi Province, Jiangxi Normal University, Nanchang 330022, China

**Keywords:** *Ceratophyllum demersum* L., response surface methodology, α-glucosidase inhibitors, phytochemical profile

## Abstract

*Ceratophyllum demersum* L. (CDL) is a traditional Chinese herb to treat many diseases, but research on its anti-diabetic activity is not available. In this research, the α-glucosidase inhibitory ability and phytochemical constituents of CDL extract were firstly studied. Optimal ultrasound-assisted extraction conditions for α-glucosidase inhibitors (AGIs) were optimized by single factor experiment and response surface methodology (RSM), which was confirmed as 70% methanol, liquid-to-solid ratio of 43 (mL/g), extraction time of 54 min, ultrasonic power of 350 W, and extraction temperature of 40 °C. The lowest IC_50_ value for α-glucosidase inhibition was 0.15 mg dried material/mL (mg DM/mL), which was much lower than that of acarbose (IC_50_ value of 0.64 mg DM/mL). In total, 80 compounds including 8 organic acids, 11 phenolic acids, 25 flavonoids, 21 fatty acids, and 15 others were identified or tentatively identified from CDL extract by HPLC-QTOF-MS/MS analysis. The results suggested that CDL could be a potential source of α-glucosidase inhibitors. It can also provide useful phytochemical information for research into other bioactivities.

## 1. Introduction

α-Glucosidase is a vital carbohydrate hydrolase situated in the brush border surface membrane of the small intestine, which is involved in the last step of carbohydrate digestion by hydrolyzing the α-(1,4) glycosidic bond to release glucose at the non-reducing end [1]. α-Glucosidase inhibitors (AGIs) can effectively alleviate the release of glucose from dietary carbohydrates and delay the absorption of glucose by inhibiting the action of α-glucosidase, resulting in delayed postprandial blood glucose level [2]. Currently, acarbose, miglitol, and voglibose are the commonly used AGIs to treat diabetes and its complications, but these drugs exhibit toxic side effects, such as flatus, diarrhea, abdominal colic, and so on [3]. At present, numerous studies have proved that many plant extracts possess the potential to be excellent sources of AGIs, with the advantages of being natural, highly-efficient, inexpensive, and with low toxicity. Moreover, many highly active AGIs have been isolated and identified, such as flavones, phenolic acids, alkaloids, terpenes, anthocyanins, glycosides, and so on [4,5]. Zhang et al. [6] evaluated the α-glucosidase inhibitory activity of four *Acer* species leaves, and the IC_50_ values were 167–433 fold of that of acarbose; hydrolysable tannins were the major contributors. 3-Caffeyl-5-feruloylquinic acid was found to be the major AGI of *Artemisia selengensis* Turcz root [7]. *Datura stramonium* L. (*Solanaceae*) root extracts [8] and *Ocimum gratissimum* leaf extracts [9] were found to show considerable potential to control the blood glucose level of streptozocin-induced diabetic rats.

At present, the conventional extraction techniques used to extract active substances from plants are mainly solvent extraction and supercritical fluid extraction [10]. Solvent extraction takes a long time to soak, and the extraction efficiency is not high. Supercritical fluid extraction requires expensive equipment and can clog the system when water is present in the sample [11]. As an emergent non-thermal extraction technology, ultrasonic-assisted extraction (UAE) is cheap and easy to use in practice [12]. In addition, it has many physical effects on the plant materials, such as fragmentation, corrosion, ultrasonic capillary effect, acoustic pore effect, and local shear, which can reduce the particle size, increase the surface area, and destroy the cell junction structure of the plant matrix, leading to improved mass transfer efficiency and enhanced extraction rate [13]. UAE is usually performed at lower temperatures to prevent thermal degradation of bio-active compounds in the extract [14]. In addition, the recovery efficacy of active components from plants is usually influenced by many factors, such as liquid-to-solid ratio, temperature, time, ultrasonic power, and solvent polarity [15]. Therefore, in order to improve extraction efficiency, reduce extraction costs, and to obtain the most active substances, it is necessary to optimize the extraction conditions. Response surface method (RSM), a mathematical and statistical tool, is widely used to optimize the extraction process, and can elucidate the possible interactions between experimental variables in various processes, reduce experiment numbers and improve statistical interpretation [16]. Zerajic et al. [17] optimized the factors (extraction time, ethanol concentration, and extraction temperature) affecting the UAE of *Calendulae officinalis* L. flowers using a Box-Behnken design (BBD). Yang et al. [18] applied the BBD to optimize the factors (methanol concentration, extraction temperature, and liquid-to-solid ratio) of the UAE of kinsenoside compound from *Anoectochilus roxburghii* (Wall.) Lindl.

*Ceratophyllum demersum* L. (CDL), also known as hornwort, is a perennial submerged macrophyte commonly found in ponds, lakes, and streams. It has been traditionally used in the treatment of diarrhea, fever, wounds, hemorrhoids or piles, intrinsic hemorrhages, hyperdipsia, and hematemesis [19]. Some studies have shown that CDL extracts showed a variety of biological activities, including antioxidant [20], antifungal [21], insecticidal [22], anti-diarrhea, and wound healing [19]. Various flavonoids like tricin-7-*O*-β-d-glucoside, naringenin-7-*O*-β-d-glucoside, apigenin-7-*O*-glucoside, and apigenin diglycoside could be the active ingredients in CDL [23,24]. However, research on the hypoglycemic effects and related active constituents of CDL are not available.

This research optimized the extraction condition of α-glucosidase inhibitors (AGIs) from CDL using RSM and characterized its phytochemical constituents. A suitable solvent for extraction of AGIs was firstly screened by determination of α-glucosidase inhibitory ability, total phenolic content, and total flavonoid content. Methanol extract (70%) was found to show the best suppression with the lowest IC_50_ value of 0.17 mg DM/mL, which was 3.7 times higher than acarbose (IC_50_ value of 0.76 mg/mL). Then, the optimal extraction conditions of AGIs in CDL were optimized by using single factor experiments and RSM. The major phytochemical components which gave the best inhibition of the activity of α-glucosidase were identified or tentatively identified by HPLC-QTOF-MS/MS.

## 2. Results and Discussion

### 2.1. Effect of Solvent Polarity on the Recovery of AGIs

**Figure 1:**
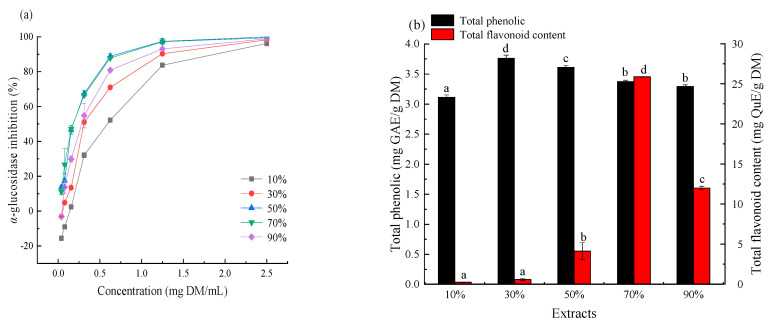
α-Glucosidase inhibition (**a**), total phenolic and total flavonoid content (**b**), of *Ceratophyllum demersum* L. (CDL) extracts prepared with different concentrations of methanol aqueous solvent.

The recovery of bioactive compounds varied greatly with the changes of solvent polarity. Therefore, the influence of different concentrations of methanol on the extraction of AGIs from CDL was evaluated separately; the result is given in Figure 1. All extracts had considerable α-glucosidase inhibition in the sample concentration range of 0.17–2.5 mg DM/mL and exhibited an obvious dose—effect relationship. The 70% methanol extract possessed the best α-glucosidase inhibition with the lowest IC_50_ value of 0.17 mg DM/mL. The inhibition was 3.7 times higher than acarbose (0.76 mg/mL), a clinical diabetes treatment drug, indicating the hypoglycemic potential of CDL extracts (Figure 1a). Analysis of total phenolic content (TPC) and total flavonoid content (TFC) indicated that 30% methanol extract possessed the highest TPC, with the value of 3.76 mg GAE/g DM. The highest TFC was found in 70% methanol extract (27.88 mg quercetin equivalents per gram of dried material (mg QuE/g DM,)). The 10% methanol extract possessed the lowest TPC and TFC, which were only 3.11 mg gallic acid equivalents per gram of dried material (mg GAE/g DM) and 0.23 mg QuE/g DM, respectively (Figure 1b). These indicated that the medium polar solvent is more suitable for extracting phenols in CDL, and the weak polar solvent is suitable for extracting flavonoids. Correlation coefficient analysis (Table S1) revealed that the flavonoids in CDL correlated well (r = −0.648) with the α-glucosidase inhibition, so flavonoids could be the major contributor to the α-glucosidase inhibition of CDL. Thus, 70% methanol was selected for further extraction of AGIs from CDL.

### 2.2. Preliminary Screening of Each Single Factor Analysis

**Figure 2:**
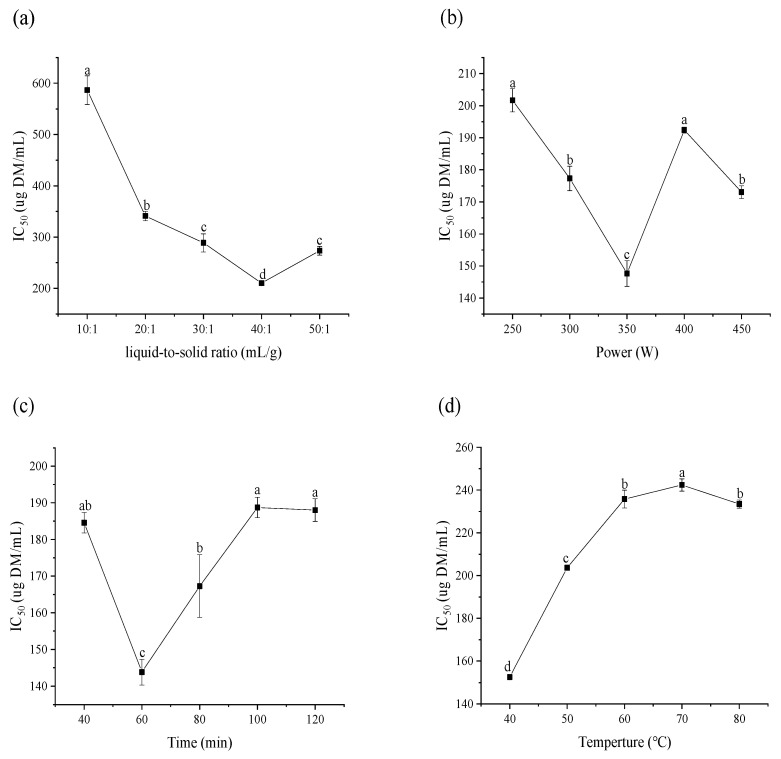
Effects of liquid-to-solid ratio (**a**), ultrasonic power (**b**), extraction time (**c**), extraction temperature (**d**), on the α-glucosidase inhibitory ability (IC_50_) of CDL extracts.

Extraction temperature, time, ultrasound power and liquid-to-solid ratio also played an important role in the recovery of bioactive constituents. Generally, the more solvents, the higher mass transfer efficiency and extraction rate, but too many solvents cause solvent waste and increase the extraction cost [25]. As shown in Figure 2a, the IC_50_ value of extracts decreased with increasing liquid-to-solid ratio with the minimum value detected at 40 mL/g, but a slight increment was observed when the ratio was set at 50 mL/g. In Figure 2b, increased ultrasonic power (250–350 W) resulted in increased α-glucosidase inhibition of extracts. Further increasing ultrasonic power resulted in reduced α-glucosidase inhibition. Therefore, 350 W was considered to be the optimal ultrasound power due to the highest α-glucosidase inhibition and relatively low energy consumption. Reasonable extraction time can facilitate the contact between solvent and raw material, which is beneficial to the release of target compounds, and increase the extraction rate [26], but continuous heating is not conducive for retention of activity. As shown in Figure 2c, the sample extracted for 60 min gave the strongest α-glucosidase inhibition.

With the increase of extraction temperature from 40 °C to 70 °C, a significant increase in IC_50_ value was observed; the minimum α-glucosidase inhibition was detected at 70 °C (Figure 2d). Usually, a higher extraction temperature can destroy cell structure more effectively, leading to increased extraction yield [27]. However, low temperature (40 °C) is more conducive to the extraction of α-glucosidase inhibitors from CDL, therefore, 40 °C was selected as the suitable extraction temperature.

### 2.3. Response Surface Analysis

Based on the results of single factorial experiments, liquid-to-solid ratio, ultrasonic power, and extraction time were chosen for further RSM analysis. The experiments were performed according to Box—Behnken design (BBD), and results are presented in Table 1. The results indicate the effect of process variables on the α-glucosidase inhibition of CDL extracts. Estimated regression coefficients for the response (IC_50_ value) in the second order polynomial equations (Equation (1)) are as follows:

Y = 146.58 − 5.05A + 1.68B + 11.11C + 12.60A^2^ + 6.44B2 + 22.54C^2^ + 0.48AB + 8.54AC − 2.21BC (1)

ANOVA statistics (Table 2) were generated to assess the goodness of fit, the significance of the model, coefficient of determination, and related probability values (*p*-value) [10]. The overall quadratic model, individual and interaction effects of liquid-to-solid ratio (mL/g), ultrasonic power (W), extraction time (min) are indicated by F and *p*-values. The *p*-value (<0.0001) showed that the model was statistically significant. At the same time, the values of R^2^ and Adj-R^2^ were 0.9798 and 0.9538, respectively, implying a strong correlation between the predicted results and actual results. Moreover, the linear effect of liquid-to-solid ratio, extraction time, and square effect of liquid-to-solid ratio, extraction power, and extraction time, were found to be significant for α-glucosidase inhibitory activity. The interaction terms of liquid-solid ratio and time have a significant effect on α-glucosidase inhibitory activity.

**Figure 3:**
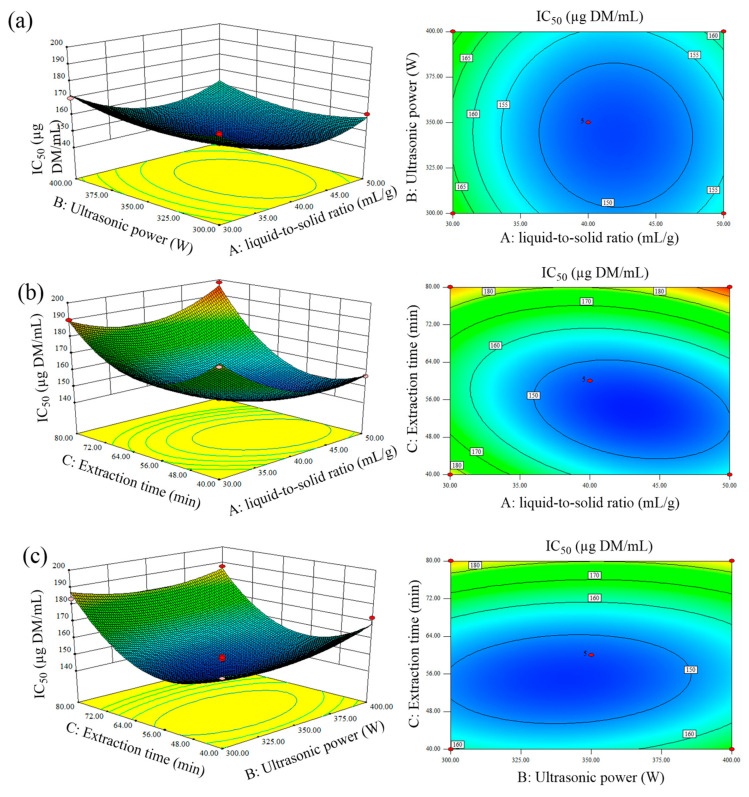
3D surface plot and contour map showing the interaction effects of (**a**) liquid-to-solid ratio and power, (**b**) liquid-to-solid ratio and time, (**c**) time and power on IC_50_.

The interaction effects of individual process variables on dependent variable (IC_50_ value) were clearly studied through the pictorial representation in the form of 3D plot and 2D contour map (Figure 3). Figure 3a illustrates that there was no significant interaction between ultrasonic power and liquid-to-solid ratio. At any liquid-to-solid ratio, the α-glucosidase inhibitory activity increased with improved ultrasonic power. As revealed by Figure 3b, when the ultrasonic power was set at 350 W, the IC_50_ value decreased by simultaneous increase of liquid-to-solid ratio and extraction time. A higher α-glucosidase inhibition was obtained when the extraction time and liquid-solid ratio reached 53 min and 43 mL/g, respectively, which implied a significant interaction between the two parameters. In Figure 3c, within the scope of 40–54 min and 300–341 W, the inhibition ability of α-glucosidase increased with the sonication time and power increase, then decreased when beyond this range. According to the significance of regression coefficients, it was evident that extraction time was the most significant factor affecting the inhibitory activity, followed by liquid-to-solid ratio and ultrasonic power.

### 2.4. Optimal Extraction Conditions Analysis

To obtain the maximized response of α-glucosidase inhibition, a response optimizer tool was used to determine the optimal level of the chosen variables. The lowest IC_50_ value of 143.88 µg DM/mL was predicted at the optimal conditions of liquid-to-solid ratio of 43 mL/g, extraction time of 54 min, and power of 340 W. Validation experiments for the predicted optimum conditions were carried out to verify the model accuracy. However, due to the limitations of actual operating conditions, the actual parameter of each variable was adjusted to 43 (mL/g), 54 min, 350 W. The experimental IC_50_ value was observed to be 146.23 µg DM/mL, which fitted well (98.37%) with the predicted IC_50_ value. This demonstrates that the developed RSM model is practicable and can be used to describe the relationship between extraction factors and α-glucosidase suppression of CDL extracts. 

### 2.5. Analysis of Phytochemical Constituents

**Figure 4:**
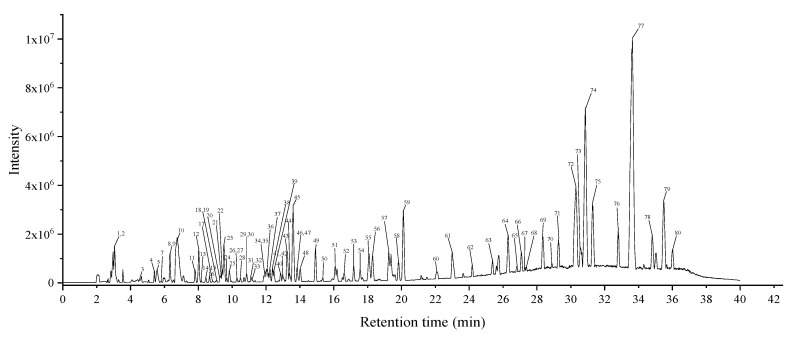
The base peak chromatogram of CDL extract under negative mode.

To investigate the major chemical components of the CDL extract giving the strongest α-glucosidase inhibition, HPLC-QTOF-MS/MS analysis was carried out. The base peak chromatogram (BPC) is shown in Figure 4. Identified or tentatively identified compounds are listed in Table 3; identities were confirmed by analyzing the fragmentation pattern of each deprotonated molecule, and by matching the data with that recorded in available references and databases. In total, 80 compounds were identified or tentatively identified, including 8 organic acids, 11 phenolic acids, 25 flavonoids, 21 fatty acids, and 15 other compounds.

#### 2.5.1. Organic Acids

**Figure 5:**
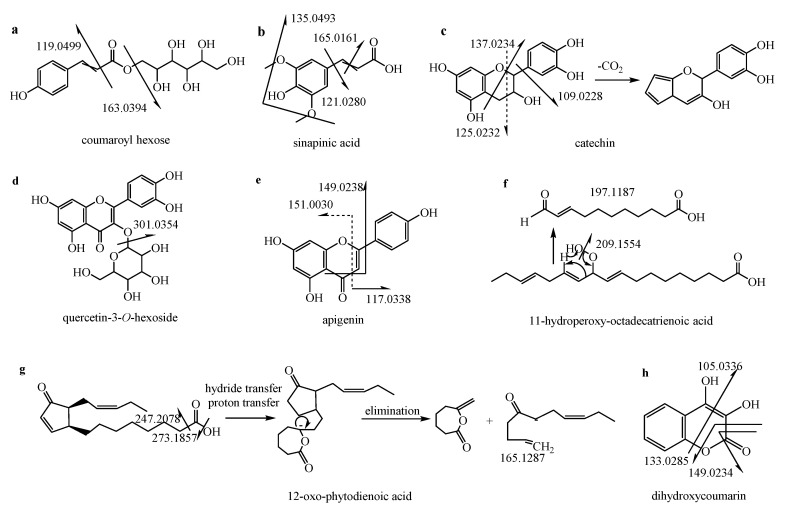
Possible fragmentation pattern of coumaroyl hexose (**a**), sinapinic acid (**b**), catechin (**c**), quercetin-3-O-hexoside (**d**), apigenin (**e**), 11-hydroperoxy octadecatrienoic acid (**f**), 12-oxo-phytodienoic acid (**g**), and dihydroxycoumarin (**h**).

A total of 8 organic acids were identified or tentatively identified in CDL extracts. Under negative ion mode, organic acids often show diagnostic fragment ions by losing H_2_O (18 Da), CO (28 Da), CO_2_ (44 Da), and HCOOH (46 Da). Peak 3 (195.0514, C_6_H_12_O_7_) was identified as gluconic acid according to reference [28]. Peaks 5, 10, 18, and 49 were individually identified as malic acid (133.0148, C_4_H_6_O_5_), citric acid (191.0200, C_6_H_8_O_7_), *p*-coumaric acid (163.0404, C_9_H_8_O_3_), and azelaic acid (187.0984, C_9_H_16_O_4_) due to the diagnostic MS/MS fragment ions at 115.002 [M − H − H_2_O]^−^, 111.0083 [M − H − CO_2_ − 2H_2_O]^−^, 119.0487 [M − H − CO_2_]^−^, and 125.0970 [M − H − C_2_H_2_O_2_]^−^, respectively [29,30]. Peaks 19, 20, and 24 (C_15_H_18_O_8_) showed the similar [M − H]^−^ at 325.0938 and similar fragmentation pattern, suggesting they were isomers. They were proposed as coumaroyl hexose and its isomers according to the fragment of [coumaric acid − H]^−^, [M − H − hexose − CO_2_]^−^, and [M − H − hexose − H_2_O]^−^ [31]. The detected fragmentation pattern of peak 19 is shown in Figure 5a.

#### 2.5.2. Phenols and Derivatives

A total of 11 phenolic acids were characterized, which can be further classified into hydroxybenzoic acids and their derivatives.

Three hydroxybenzoic acid derivatives were identified. Peak 9 (329.0878, C_14_H_18_O_9_) was tentatively characterized as vanilloyl glucoside due to the fragment ions at *m/z* 167.0341 [vanillic acid − H]^−^, 152.0120 [M − H-glucose − CH_3_]^−^, and 123.0438 [M − H − glucose − CO_2_]^−^ [32]. Peaks 26 (183.0307, C_8_H_8_O_5_) and 42 (197.0455, C_9_H_10_O_5_) had similar fragment ions at 124.01 (C_6_H_4_O_3_), their molecular weights were 14 and 28 Da higher than gallic acid, respectively, corresponding to the augment of one and two methylene. Diagnostic MS/MS ions at 169.0138 [gallic acid − H]^−^ and 125.0235 [M − H − gallic acid − CO_2_]^−^ revealed the assignment of methyl gallate and ethyl gallate, respectively [6,33].

Eight hydroxycinnamic acids were identified, including caffeic acid derivatives, sinapinic acid and its derivatives, ferulic acid and its derivatives. Peaks 11 and 16 were tentatively confirmed as caffeoyl hexose (341.0884, C_15_H_18_O_9_) by the diagnostic MS/MS fragment ions at 179.0344 [caffeic acid − H]^−^ [32]. The fragment ions of peak 14 at 208.0322, 193.0161, and 149.0253 resulting from the loss of CH_3_, 2 CH_3,_ and 2 CH_3_ + COOH respectively, indicating the presence of two methyl groups and one propenoic acid moiety. So it was identified as sinapinic acid [34], and the detected fragmentation pattern is given in Figure 5b. Peaks 15 and 25 gave the same parent ion ([M − H]^−^ of 385.11, C_17_H_22_O_10_) and product ions were identified as sinapoylglucose and its isomer [32]. MS/MS ions at 223.06, 179.07, 164.05, 149.02 resulted from the successive breakage of glucose, CO_2_, and CH_3_, and CH_3_. Peak 47 (193.0507, C_10_H_10_O_4_) with the MS/MS ions at 178.0253 [M − H − CH_3_]^−^, 134.0368 [M − H − CH_3_ − CO_2_]^−^, and 133.0287 [M − H − C_4_H_4_O_2_]^−^ was identified as ferulic acid [35]. Then peak 13 (C_22_H_30_O_14_) with *m/z* at 517.1584 was tentatively confirmed as feruloyl sucrose due to the fragment ion at 193.0506 [ferulic acid − H]^−^ [31]. Similarly, peak 23 (355.1052, C_16_H_20_O_9_) was identified as feruloyl glucose [32].

#### 2.5.3. Flavonoids

In total, 25 flavonoids were found in CDL, such as quercetin, kaempferol, naringenin, apigenin, catechin, and their derivatives. Currently, tricin-7-*O*-β-d-glucoside, naringenin-7-*O*-β-d-glucoside, and apigenin-7-*O*-glucoside have been identified from CDL.

Apigenin, quercetin, kaempferol, naringenin, luteolin, myricetin, laricitrin, syringetin, chrysoeriol, and catechin have the typical aglycone ion (Y_0_^-^) at 269.04, 301.03, 285.04, 271.06, 285.04, 317.03, 331.05, 345.06, 299.05, and 289.07, respectively. Consequently, their derivatives will exhibit corresponding characteristic aglycone ions by losing glycoside moiety, e.g., pentosyl (132 Da), glucosyl (162 Da), hexosyl (162 Da), rhamnosyl (146 Da) or rutinosyl (308 Da). Under negative ion mode, flavonoids will exhibit typical losses of CO, CO_2_, C_3_O_2_, and C_2_H_2_O. Flavones are more likely to produce ions at ^1,3^A^−^ and ^1,3^B^−^, and flavonols are easier to get fragment ions at ^1,2^A^−^ and ^1,2^B^−^ [36,37]. In addition, when the glycosidic bond is bonded to the 3-OH position of aglycone, Y_0_^−^ and [Y_0_ − H]^−^ fragments will occur, but the intensity of [Y_0_ − H]^−^ is customarily higher than that of Y_0_^−^ [38].

For instance, peak 17 (577.1378, C_30_H_26_O_12_) was tentatively identified as procyanidin dimmer due to the diagnostic ion at 289.0720 [(Epi) catechin − H]^−^ [30]. Peaks 22 and 27 with the same deprotonated ion at 289.07 and MS/MS fragment ions at 245.08 [M − H − CO_2_]^−^, 137.02 [^1,3^A]^−^, 125.02 [^1,4^A]^−^, and 109.02 [B-ring − H]^−^ were identified as (epi) catechin by comparing the data with those reported in reference [6]. The detected fragmentation pattern of peak 22 was shown in Figure 5c. Peaks 28, 36, and 48 with [M − H]^−^ at 463.09 were ascribed to quercetin-3-*O*-hexoside, the coexist of aglycone ion 301.03 and deprotonated ion 300.03 indicating the attachment of hexoside to the 3-OH [39]; the detected fragmentation pattern of peak 28 is shown in Figure 5d. Peak 32 (609.1495, C_27_H_30_O_16_) with fragment ion at 301.0357 resulted from the loss of rutinosyl (308 Da), thus it was identified as quercetin-3-*O*-rutinoside [30]. Peak 50 was identified as quercetin due to the aglycone ion at 301.0367 and MS/MS ion at 151.0027 [^1,3^A]^−^. In the same way, peak 33 (593.1549, C_27_H_30_O_15_) was characterized as kaempferol-3-*O*-rutinoside due to the aglycone ion at 285.05 [30]. Peaks 41 and 46 yielded deprotonated ions at *m/z* 447.09 (C_21_H_20_O_11_), and product ions at *m/z* 285.05 were tentatively identified as kaempferol-3-*O*-hexoside [40]. Peak 51 (287.0565, C_15_H_12_O_6_) had two more hydrogen atoms compared with kaempferol; fragment ions at 259.0611 [M − H − CO]^−^, 151.0028 [^1,3^A]^−^, 125.0239 [^1,4^A]^−^ allowed the assignment of dihydrokaempferol. Analogously, apigenin (peak 58) and its glycosides (peaks 21, 37, 43), luteolin (peak 54) and luteolin-7-*O*-hexoside (peak 34), naringenin (peak 59) and its glycosides (peaks 38, 45), myricetin-3-*O*-hexoside (peak 30), laricitrin-3-*O*-hexoside (peak 35), syringetin-3-*O*-hexoside (peak 40), chrysoeriol-7-*O*-hexoside (peak 44) were proposed by matching the MS and MS/MS data with those recorded in the literature and databases [29,35,41,42]. The detected fragmentation pattern of peak 58 is shown in Figure 5e.

#### 2.5.4. Fatty Acids

In total, 21 fatty acids were found in CDL extracts. Peaks 55 and 56 exhibited precursor ions [M-H]^−^ at *m/z* 327.22. Product ions at 291.19 and 229.14 resulted from the successive loss of 2H_2_O and 3H_2_O + CO_2_, indicating the existence of 3 hydroxy groups and one carboxyl group. Thus, they were tentatively characterized as trihydroxy octadecadienoic acid [30]. Peaks 57 and 70 (329.23, C_18_H_34_O_5_) were tentatively characterized as trihydroxy octadecenoic acid due to a mass difference of 2 amu with peak 55. Moreover, five isomers of hydroperoxides of octadecatrienoic acid (peaks 60, 64, 65, 66, and 68, *m/z* at 309.21, C_18_H_30_O_4_) and three isomers of hydroperoxides of octadecadienoic acid (peaks 67, 69, and 71, *m/z* at 311.22, C_18_H_32_O_4_) were found. In general, isomers can be distinguished by diagnostic ions, hydroperoxy-linoleic acid isomers with product ions at 223 [M − H − C_4_H_6_O − H_2_O]^−^, 183 [M − H − C_7_H_12_O_2_]^−^, 171 [M − H − C_9_H_14_ − H_2_O]^−^ or 211 [M − H − C_6_H_12_O]^−^, while hydroperoxy-linolenic acid isomers with characteristic ions at 251 [M − H − C_3_H_5_ − H_2_O]^−^, and 197 [M − H − C_7_H_11_ − H_2_O]^−^ helped to assign the position of the hydroperoxide [28]. Taking peak 65 as an example, the diagnostic fragment ion at 197.12 suggested the presence of a hydroperoxide at C11, so it was identified as 11-hydroperoxy-octadecatrienoic acid; the fragmentation pattern is shown in Figure 5f. Peaks 60, 64, and 66 with product ions at 171.10 resulted from the loss of C_9_H_14_O, indicating the hydroperoxide at C9, but this could not reveal the position of the double bonds. Peaks 67, 69, and 71 were identified as 9-hydroperoxy -octadecadienoic acid due to the MS/MS at 171.10.

Peak 72 with molecular ion at *m/z* 291.1980 was identified as 12-oxo-phytodienoic acid, and the fragmentation pattern is shown in Figure 5g. Its MS/MS ions at 273.1857 and 247.2078 result from the loss of a water molecule and a carboxylic residue, respectively [43]. Peak 73 (559.3142, C_28_H_48_O_11_) was tentatively assigned as dirhamnosyl linolenic acid, fragment ion at 277.2186 resulted from the loss of a dirhamnosyl (C_10_H_18_O_9_, 282 Da) [36].

In addition, five peaks with similar [M − H]^−^ at 293.21 (C_18_H_30_O_3_) were detected. Peaks 74 and 75 with diagnostic fragment ions at 171.1032 and 195.1387 were identified as hydroxy octadecatrienoic acid [44], while peaks 78, 79, and 80 with characteristic ions at 113.09 or 185.11 were identified as oxo-octadecadienoic acid [45]. Analogously, peak 76 (291.1977, C_18_H_28_O_3_) was tentatively proposed as oxo-octadecatrienoic acid [28]. Peak 77 (295.2283, C_18_H_32_O_3_) was proposed as 9-hydroxy-10,15-octadecadienoic acid due to the MS/MS ions at 277.2158 [M − H − H_2_O]^−^, 195.1387 [M − H − C_6_H_12_O]^−^, and 171.1026 [M − H − C_9_H_16_]^−^ [36].

#### 2.5.5. Others

Another 15 compounds belonging to other category were also detected. Two saccharides (peaks 1 and 2) were tentatively identified due to the characteristic fragment ions at 179.0595 [M − H − C_6_H_10_O_5_]^−^ [32] and 113.0234 [M − 2H_2_O − CH_2_OH]^−^ [46]. Peak 6 (137.0247, C_7_H_6_O_3_) was tentatively characterized as protocatechualdehyde [30]. Peak 29 (177.0204, C_9_H_6_O_4_) was detected as dihydroxycoumarin [47], MS/MS ions at 149.0234, 133.0285, and 105.0336 individually corresponded to the loss of CO, CO_2_, and C_2_O_3_; the detected possible fragmentation pattern is given in Figure 5h. In a similar way, peaks 31 (431.1938, C_20_H_32_O_10_), 53 (201.1145, C_10_H_18_O_4_), 61 (307.1928, C_18_H_28_O_4_), and 62 (311.1878, C_17_H_28_O_5_) were tentatively identified as hydroxy-2,4,4-trimethyl-3-(3-oxobutyl)-2-cyclohexen-1-one glucoside, dibutyl oxalate, dihydrocapsiate, and dihydroartemisinin ethyl ether, respectively, by matching the MS and MS/MS data with those recorded in reference [6,30,31]. Peaks 4, 7, 8, 12, 39, 52, and 63 were not identified due to the lack of MS/MS information.

## 3. Material and Methods

### 3.1. Reagents

Acarbose, *p*-nitrophenyl-α-d-glucopyranoside (*p*NPG), α-glucosidase (yeast, EC 3.2.1.20), Folin-Ciocalteu reagent were from Sigma-Aldrich (Sigma, St. Louis, MO, USA). All other used reagents were of analytical grade and purchased from Aladdin (Shanghai, China).

### 3.2. Preparation of Extracts

Fresh CDL was bought in Shuyang County, Jiangshu Province, in April 2019. The CDL was dried, pulverized into powder with a high-speed disintegrator (Hangzhou, China), and sieved through a 50 mesh screen. The plant material′s moisture content was 8.2% (*w/w*), which was determined by measuring the weight before and after drying at 105 °C in a bake oven to a constant weight. The CDL powder was stored in a refrigerator at −20 °C until used.

Selecting a suitable solvent is very important for extracting the target product. In this research, a methanol solution was selected as the best extraction solvent after pre-experiment. The CDL powder (1 g) was suspended in 10%, 30%, 50%, 70%, and 90% methanol aqueous solution at a liquid-to-solid ratio of 20 mL/g, respectively, and then sonicated for 120 min at 50 °C, 200 W. The mixtures were centrifuged at 5000 rpm/min for 10 min, and the supernatants were collected for further analysis.

### 3.3. Determination of Total Phenolic and Flavonoid Content

The total flavonoid content (TFC) and total phenolic content (TPC) of different crude extracts were measured with the AlCl_3_ colorimetric method and the Folin—Ciocalteu method [48] with some modifications, respectively. In the experiment of measuring TFC, 0.5 mL of properly diluted sample was mixed with 100 μL of 5% NaNO_2_ for 6 min, followed by adding 100 μL 10% AlCl_3_ for 6 min, then adding 1 mL 4% NaOH and 1 mL distilled water. The mixtures were incubated at room temperature for 15 min, and 200 μL of mixtures were pipetted into a 96-well plate. The absorbance was measured at 510 nm using a microplate reader (SpectraMax M2, Molecular Devices Corp., Sunnyvale, CA, USA). In the experiment of measuring TPC, 200 μL of properly diluted sample was incubated with 100 μL of Folin—Ciocalteu reagent for 5 min, followed by adding 300 μL 20% Na_2_CO_3_ and 1 mL distilled water. The mixtures were incubated at room temperature for 30 min in the dark. After 2 min of centrifugation at 7000 rpm, 200 μL of supernatants were pipetted into a 96-well plate, and absorbance at 765 nm was read with a micro-plate reader. The TFC was expressed as mg quercetin equivalents per gram of dried material (mg QuE/g DM). The TPC was expressed as mg of gallic acid equivalents per gram of dried material (mg GAE/g DM.). All experiments were done in triplicate.

### 3.4. Single Factor Experiments

The liquid-to-solid ratio, ultrasonic power, extraction time, and extraction temperature were the major factors affecting the recovery of bioactive compounds from plant materials. The experiments were performed by changing the level of one factor and maintaining the other factors at a constant level of 70% methanol aqueous solvent, liquid-to-solid ratio at 40 mL/g, extraction time at 60 min, ultrasonic power at 300 W, and extraction temperature at 50 °C. Briefly, CDL was extracted with 70% methanol aqueous solvent in different liquid-to-solid ratios (from 10 to 50 mL/g) at different extraction times (from 40 to 120 min), ultrasonic powers (from 250 to 450 W), and temperatures (from 40 to 80 °C) controlled by a digitally-controlled ultrasonic bath (KQ-500DE, Kunshan ultrasonic instrument CO., LTD, Kunshan, China).

### 3.5. *α-*Glucosidase Inhibition Assay

The α-glucosidase inhibition was assessed using the method reported by reference [6]. All α-glucosidase and *p*NPG solutions were prepared with 0.1 M, pH 6.9 phosphate buffer. Different concentrations of samples (50 μL) and 50 μL of 0.1 U/mL α-glucosidase solution were incubated in 96-well plates at 25 °C for 10 min. Then, 50 μL of 5 mM *p*NPG solution was added and incubated for 15 min at 37 °C. Finally, the reaction was terminated with 100 μL of 0.2 M Na_2_CO_3_, and absorbance at 405 nm was recorded with a micro-plate reader. Acarbose was used as positive control. All experiments were done in triplicate. The concentration required to inhibit 50% activity of α-glucosidase (IC_50_ value) was expressed as mg dried material/mL (mg DM/mL).

### 3.6. Statistical Optimization of UAE

RSM with BBD was used to optimize the extraction of AGIs in CDL. As shown in Table 4, three extraction variables (ratio of material to liquid: 1:30, 1:40, and 1:50 g/mL; extraction time: 40, 60, and 80 min; ultrasonic power: 300, 350, and 400 W) were chosen to evaluate the effect on response value (α-glucosidase inhibitory ability). The response variables were fitted to the following, a second order polynomial model equation:

(2) $$Y=\alpha_{0}+\sum_{i=1}^{k}\alpha_{ii}X_{ii}^{2}+\sum_{i}^{k-1}\sum_{j}^{k}\alpha_{ij}X_{i}X_{j}$$

where Y is the predicted response value (α-glucosidase inhibitory ability); X_i_ and X_j_ are independent variables; α_0_, α_i_, α_ii_, and α_ij_ are the constant coefficient, linear coefficient, quadratic coefficient, cross-product coefficient, respectively.

### 3.7. HPLC-QTOF-MS/MS Analysis

For compound separation, an Agilent 1260 HPLC infinity system (Agilent, Palo Alto, CA, USA) equipped with a DAD detector, a binary pump, and a Sun^Fire^ C_18_ column (250 × 4.60 mm, 5 μm, Waters, Milford, MA, USA) was applied. The mobile phase consisted of 0.1% formic acid in de-ionized water (A) and acetonitrile (B). The sample was eluted with a gradient from 10% B to 100% B in 35 min at a flow rate of 0.8 mL/min. The detection wavelength, column temperature, and injection volume were set at 280 nm, 35 °C, and 5 μL, respectively.

To obtain the MS and MS/MS information of detected compounds, the elutes were directly interfaced to a Hybrid Quadrupole-TOF 6600 system (AB Sciex) equipped with an electrospray ionization source (ESI). The full scan mass spectrum was detected at a mass range of *m/z* 100–1500 under negative ion mode. Other parameters were spray gas pressure of 50 psi, capillary voltage of 3.5 kV, ion source temperature of 550 °C, flow rate of 0.8 mL/min, and ion spray voltage floating of − 4500 V. Nitrogen and helium were used as auxiliary and collision gases, respectively. The MS data was processed by MassHunter. A molecular formula calculator was used to calculate the elemental composition of each parent and product ion. The compounds were characterized or tentatively characterized by comparing the parent ion and MS^2^ fragments with those in references and database.

### 3.8. Statistical Analysis

Statistical analyses were carried out on SPSS 17.0 (IBM, Armonk, NY, USA) and Origin 8.0 (OriginLab, Northampton, MA, USA), all data were expressed as mean ± SD (standard deviation). The statistical analysis of the proposed regression model was analyzed by Design Expert 8.0.6 (Stat-ease INC., Minneapolis, MN, USA). Significant difference among data was performed by Tukey’s-b, One-way analysis of variance (ANOVA), *p* < 0.05 was considered significant. The correlation between the bioactivity and content of constituents was evaluated by Pearson’s correlation analysis.

## 4. Conclusions

This is the first research to optimize the extraction conditions of AGIs from CDL, and to analyze the major phytochemical constituents. The optimal extraction parameters were confirmed as extraction solvent of 70% methanol, liquid-to-solid ratio of 43 (mL/g), extraction time of 54 min, ultrasonic power of 350 W, and extraction temperature of 40 °C, under which, the strongest α-glucosidase inhibition ability was achieved (IC_50_, 146.23 µg DM/mL). In addition, 30% and 70% methanol aqueous solutions are suitable for recovering the phenolics and flavonoids in CDL, respectively. HPLC-QTOF-MS/MS analyses permitted the identification of 80 compounds, including flavonoids, phenolic acids, fatty acids, and others. The major active compounds in CDL extract are caffeic acid derivatives, ferulic acid and its derivatives, apigenin, quercetin, kaempferol, naringenin, luteolin, and catechin and their derivatives, many of which have been reported to be promising AGIs. In addition, fatty acids with 18 carbons were also identified as the main components. This study can provide a theoretical basis for the study of CDL as a natural anti-diabetic drug, and the structure and inhibition mechanism of AGIs from CDL need further study.

## Figures and Tables

**Table 1:** Box—Behnken design for extraction of α-glucosidase inhibitors (AGIs) from CDL by ultrasonic-assisted extraction (UAE) with the observed responses.

Std no	A: Liquid-to-Solid Ratio (mL/g)	B: Power (W)	C: Time (min)	Response: IC_50_ (µg DM/mL)
1	40:1	300	40	159.20
2	30:1	350	40	182.30
3	40:1	350	80	189.97
4	40:1	350	60	149.14
5	50:1	300	60	160.40
6	30:1	400	60	169.88
7	40:1	400	40	172.22
8	50:1	400	60	159.50
9	40:1	350	60	146.90
10	30:1	300	60	172.71
11	50:1	350	80	198.20
12	40:1	400	80	187.48
13	50:1	350	40	156.40
14	40:1	350	60	148.13
15	40:1	350	60	142.26
16	40:1	300	80	183.31
17	40:1	350	60	146.47

**Table 2:** ANOVA statistics for the α-glucosidase inhibitory activity of extracts.

Source	Sum of Squares	df	Mean Square	F Value	*p*-Value Prob > F	Significance
Model	4764.62	9	529.40	37.69	<0.0001	**
A-liquid-to-solid ratio	203.74	1	203.74	14.50	0.0066	**
B-Power	22.65	1	22.65	1.61	0.2447	
C-Time	986.78	1	986.78	70.25	<0.0001	**
A^2^	668.94	1	668.94	47.62	0.0002	**
B^2^	174.45	1	174.45	12.42	0.0097	**
C^2^	2138.47	1	2138.47	152.25	<0.0001	**
AB	0.94	1	0.94	0.067	0.8034	
AC	291.45	1	291.45	20.75	0.0026	**
BC	19.55	1	19.55	1.39	0.2767	
Residual	98.32	7	14.05			
Lack of Fit	70.57	3	23.52	3.39	0.1345	
Pure Error	27.75	4	6.94			
Total	4862.94	16				
R^2^ = 0.9798 R^2^_Adj_ = 0.9538						

Note: ** indicates significant difference at 0.01 level.

**Table 3:** The identified or tentatively identified compounds in 70% methanol extract of CDL by HPLC-QTOF-MS/MS under negative ion mode.

No.	Rt (min)	Found at *m/z*	Expected at *m/z*	Error (ppm)	Molecular Formula	MS/MS	Proposed Compounds
Organic acids							
3	4.53	195.0514	195.0510	1.9	C_6_H_12_O_7_	-	Gluconic acid
5	5.57	133.0148	133.0142	4.2	C_4_H_6_O_5_	115.002[M − H − H_2_O]^−^	Malic acid
10	6.75	191.0200	191.0197	1.4	C_6_H_8_O_7_	111.0083[M − H − CO_2_ − 2H_2_O]^−^	Citric acid
18	9.07	163.0404	163.0401	2.2	C_9_H_8_O_3_	119.0487[M − H − CO_2_]^−^	*p*-Coumaric acid
19	9.08	325.0937	325.0938	0.3	C_15_H_18_O_8_	163.0394[M − H − hexose]^−^, 119.0499[M − H − hexose − CO_2_]^−^	Coumaroyl hexose
20	9.32	325.0939	325.0942	−0.8	C_15_H_18_O_8_	145.0927[M − H − hexose − H_2_O]^−^, 117.0342[M − H − hexose − H_2_O − CO]^−^	Coumaroyl hexose
24	9.69	325.0941	325.0939	0.7	C_15_H_18_O_8_	145.0927[M − H − hexose − H_2_O]^−^, 117.0342[M − H − hexose − H_2_O − CO]^−^	Coumaroyl hexose
49	14.92	187.0984	187.0982	1.0	C_9_H_16_O_4_	125.0970[M − H − C_2_H_2_O_2_]^−^	Azelaic acid
Phenols acids and derivatives							
9	6.31	329.0879	329.0878	0.3	C_14_H_18_O_9_	167.0341[M − H − glucose]^−^, 152.0120[M − H − C_7_H_13_O_5_]^−^, 123.0438[M − H − C_7_H_10_O_7_]^−^, 108.0210[M − H − C_8_H_13_O_7_]^−^	Vanilloyl glucoside
11	7.81	341.0884	341.0883	0.4	C_15_H_18_O_9_	179.0344[M − H − hexose]^−^, 161.0244[M − H − C_6_H_12_O_6_]^−^, 133.0293[M − H − C_7_H_12_O_7_]^−^,	Caffeoyl-hexose
13	8.18	517.1584	517.1563	4.2	C_22_H_30_O_14_	193.0506[ferulic acid − H]^−^	Feruloyl sucrose
14	8.44	223.0621	223.0612	4.2	C_11_H_12_O_5_	208.0322[M − H − CH_3_]^−^, 193.0122[M − H − CH_2_O]^−^, 165.0175[M − H − C_2_H_2_COOH]^−^, 135.0440[M − H − C_3_H_4_O_3_]^−^, 121.0298[M − H − C_4_H_6_O_3_]^−^	Sinapinic acid
15	8.44	385.1159	385.1140	4.8	C_17_H_22_O_10_	223.0606[M − H − glucose]^−^, 208.0365[M − H − C_7_H_13_O_5_]^−^, 193.0154[M − H − C_8_H_16_O_5_]^−^, 179.0714[M − H − C_7_H_10_O_7_]^−^, 164.0476[M − H − C_8_H_13_O_7_]^−^, 149.0235[M − H − C_9_H_16_O_7_]^−^	Sinapoylglucose
16	8.66	341.0886	341.0881	1.1	C_15_H_18_O_9_	179.0354[M − H − hexose]^−^, 135.0449[M − H − C_7_H_10_O_7_]^−^	Caffeoyl hexose
23	9.51	355.1052	355.1035	−0.2	C_16_H_20_O_9_	193.0511[M − glucose]^−^, 178.0272[M − H − C_7_H_13_O_5_]^−^, 149.0606[M − H − C_7_H_10_O_7_]^−^, 134.0372[M − H − C_8_H_13_O_7_]^−^	Feruloyl glucose
25	9.84	385.1158	385.1140	4.7	C_17_H_22_O_10_	223.0606[M − H − glucose]^−^, 208.0365[M − H − C_7_H_13_O_5_]^−^, 193.0154[M − H − C_8_H_16_O_5_]^−^, 179.0714[M − H − C_7_H_10_O_7_]^−^, 164.0476[M − H − C_8_H_13_O_7_]^−^, 149.0235[M − H − C_9_H_16_O_7_]^−^	Sinapoylglucose
26	10.28	183.0307	183.0299	4.3	C_8_H_8_O_5_	124.0158[M − H − C_2_H_3_O_2_]^−^	Methyl gallate
42	13.05	197.0465	197.0455	5.0	C_9_H_10_O_5_	169.0138[M − H − C_2_H_4_]^−^, 125.0235[M − H − C_3_H_4_O_2_]^−^, 124.0163[M − H − C_3_H_5_O_2_]^−^	Ethyl gallate
47	13.84	193.0507	193.0506	0.3	C_10_H_10_O_4_	178.0253[M − H − CH_3_]^−^, 134.0368[M − H − CH_3_ − CO_2_]^−^, 133.0287[M − H − C_4_H_4_O_2_]^−^	Ferulic acid
Flavonoids							
17	8.85	577.1378	577.1315	4.7	C_30_H_26_O_12_	289.0720[(Epi) catechin − H]^−^	Procyanidin dimmer
21	9.32	401.1471	401.1453	4.5	C_18_H_26_O_10_	355.1037[M − H − H_2_O − CO]^−^, 269.1040[apigenin − H]^−^, 223.0582[M − H − C_7_H_14_O_5_]^−^, 161.0448[M − H − C_9_H_20_O_7_]^−^,	Apigenin pentose
22	9.41	289.0724	289.0718	2.3	C_15_H_14_O_6_	245.0782[M − H − CO_2_]^−^, 137.0234[M − H − C_8_H_8_O_3_]^−^, 125.0232[M − H − C_9_H_8_O_3_]^−^, 109.0228[B-ring − H]^−^	(Epi)catechin
27	10.28	289.0722	289.0718	1.7	C_15_H_14_O_6_	245.0782[M − H − CO_2_]^−^, 137.0234[M − H − C_8_H_8_O_3_]^−^, 125.0232[M − H − C_9_H_8_O_3_]^−^, 109.0228[B-ring − H]^−^	(Epi)catechin
28	10.50	463.0900	463.0898	0.5	C_21_H_20_O_12_	463.0898[M − H]^−^, 301.0354[M − H − hexose]^−^, 300.0280[M − H − C_6_H_11_O_5_]^−^	Quercetin-3-*O*-hexoside
30	10.85	479.0842	479.0831	2.2	C_21_H_20_O_13_	259.0262[M − H − C_8_H_12_O_7_]^−^	Myricetin-3-*O*-hexoside
32	11.12	609.1495	609.1490	0.5	C_27_H_30_O_16_	301.0357[M − H − rutinose]^−^	Quercetin-3-*O*-rutinoside
33	11.14	593.1549	593.1546	0.5	C_27_H_30_O_15_	285.0411[M − H − rutinose]^−^, 284.0320[M − H − C_12_H_21_O_9_]^−^, 151.0027[M − H − rutinose − C_8_H_5_O]^−^	Kaempferol-3-*O*-rutinoside
34	11.93	447.0960	447.0959	−0.1	C_21_H_20_O_11_	447.0963[M − H]^−^, 285.0419[M − H − hexose]^−^, 284.0336[M − H − C_6_H_11_O_5_]^−^	Luteolin-7-*O*-hexoside
35	11.93	493.1007	493.0988	3.9	C_22_H_22_O_13_	331.0465[M − H − hexose]^−^, 315.0157[M − H − C_6_H_10_O_6_]^−^	Laricitrin-3-*O*-hexoside
36	12.05	463.0901	463.0898	0.5	C_21_H_20_O_12_	301.0363[M − H − hexose]^−^, 300.0282[M − H − C_6_H_11_O_5_]^−^	Quercetin-3-*O*-hexoside
37	12.23	577.1621	577.1621	0.0	C_27_H_30_O_14_	269.0459[M − H − rutinose]^−^, 268.0375[M − H − C_12_H_21_O_9_]^−^	Apigenin-7-*O*-rutinoside
38	12.38	579.1751	579.1743	1,4	C_27_H_32_O_14_	271.0622[M − H − C_12_H_20_O_9_]^−^, 151.0035[M − H − C_20_H_28_O_10_]^−^	Naringin
40	12.86	507.1174	507.1176	−0.4	C_23_H_24_O_13_	345.0619[M − H − hexose]^−^, 344.0553[M − H − C_6_H_11_O_5_]^−^, 329.0309[M − H − C_6_H_10_O_6_]^−^, 273.0416[M − H − C_8_H_10_O_8_]^−^	Syringetin-3-*O*-hexoside
41	12.98	447.0951	447.0953	−0.4	C_21_H_20_O_11_	285.0481[M − H − hexose]^−^, 284.0339[M − H − C_6_H_11_O_5_]^−^, 227.0361[M − H − C_8_H_12_O_7_]^−^	Kaempferol-3-*O*-hexoside
43	13.25	431.1000	431.0980	3.7	C_21_H_20_O_10_	431.0983[M − H]^−^, 269.0463[M − H − glucose]^−^, 268.0388[M − H − C_6_H_11_O_5_]^−^	Apigenin-7-*O*-glucoside
44	13.38	461.1095	461.1089	1.2	C_22_H_22_O_11_	446.0876[M − H − CH_3_]^−^, 299.0553[M − H − hexoside]^−^, 298.0487[M − H − C_6_H_11_O_5_]^−^, 283.0249[M − H − C_6_H_10_O_6_]^−^, 255.0305[M − H − C_8_H_10_O_7_]^−^	Chrysoeriol-*O*-hexoside
45	13.60	433.1157	433.1140	3.8	C_21_H_22_O_10_	271.0622[M − H − glucose]^−^, 151.0029[M − H − C_14_H_18_O_6_], 119.0493[M − H − C_13_H_14_O_9_]^−^	Naringenin-7-*O*-glucoside
46	13.84	477.0959	477.0960	−0.1	C_21_H_20_O_11_	285.0412[M − H − hexoside]^−^	Kaempferol-3-*O*-hexoside
48	14.02	463.0918	463.0908	2.2	C_21_H_20_O_12_	301.0363[M − H − hexoside]^−^	Quercetin-3-*O*-hexoside
50	15.34	301.0367	301.0354	4.6	C_15_H_10_O_7_	301.0363[M − H]^−^, 151.0027[M − H − C_8_H_8_O_3_]^−^, 149.0240[M − H − C_8_H_10_O_3_]^−^	Quercetin
51	16.08	287.0565	287.0561	1.2	C_15_H_12_O_6_	259.0611[M − H − CO]^−^, 177.0553[M − H − C_5_H_4_O_3_]^−^, 151.0028[M − H − C_8_H_8_O_2_]^−^, 125.0239[M − H − C_9_H_6_O_3_]^−^	Dihydrokaempferol
54	17.56	285.0417	285.0415	0.8	C_15_H_10_O_6_	285.0414[M − H]^−^, 175.0400[M − H − C_5_H_2_O_3_]^−^, 151.0030[M − H − C_8_H_6_O_2_]^−^, 133.0297[M − H − C_7_H_4_O_4_]^−^,	Luteolin
58	19.82	269.0466	269.0456	4.0	C_15_H_10_O_5_	269.0455[M − H]^−^, 151.0030[M − H − C_8_H_6_O]^−^, 149.0238[M − H − C_7_H_4_O_2_]^−^, 117.0338[M − H − C_7_H_4_O_4_]^−^	Apigenin
59	20.11	271.0624	271.0612	4.5	C_15_H_12_O_5_	151.0030[M − H − C_8_H_8_O]^−^, 119.0499[M − H − C_7_H_4_O_4_]^−^	Naringenin
Fatty acids							
55	18.08	327.2186	327.2183	−1.3	C_18_H_32_O_5_	291.1957[M − H − 2H_2_O]^−^, 229.1442[M − H − 3H_2_O − CO_2_]^−^, 171.1030[M − H − C_8_H_12_O_3_]^−^	Trihydroxy octadecadienoic acid
56	18.29	327.2177	327.2181	4.5	C_18_H_32_O_5_	291.1971[M − H − 2H_2_O]^−^, 229.1442[M − H − 3H_2_O − CO_2_]^−^, 171.1032[M − H − C_8_H_12_O_3_]^−^	Trihydroxy octadecadienoic acid
57	19.25	329.2353	329.2351	0.7	C_18_H_34_O_5_	211.1345[M − H − C_6_H_14_O_2_]^−^, 171.1029[M − H − C_8_H_14_O_3_]^−^	Trihydroxy octadecenoic acid
60	22.09	309.2075	309.2071	1.2	C_18_H_30_O_4_	291.1973[M − H − H_2_O]^−^, 265.2159[M − H − C_3_H_8_]^−^, 171.1018[M − H − C_9_H_14_O]^−^	Hydroxy octadecatrienoic acid
64	26.29	309.2075	309.2071	1.2	C_18_H_30_O_4_	291.1973[M − H − H_2_O]^−^, 185.1188[M − H − C_8_H_12_O]^−^, 171.1031[M − H − C_9_H_14_O]^−^	Hydroxy octadecatrienoic acid
65	26.80	309.2077	309.2071	1.8	C_18_H_30_O_4_	209.1554[M − H − C_6_H_12_O]^−^, 197.1187[M − H − C_7_H_12_O]^−^	11-Hydroperoxy octadecatrienoic acid
66	27.08	309.2083	309.2071	4.0	C_18_H_30_O_4_	291.1962[M − H − H_2_O]^−^, 185.1183[M − H − C_8_H_12_O]^−^, 171.1028[M − H − C_9_H_14_O]^−^	Hydroxy octadecatrienoic acid
67	27.27	311.2240	311.2228	3.8	C_18_H_32_O_4_	293.2107[M − H − H_2_O]^−^, 185.1172[M − H − C_8_H_14_O]^−^, 171.1023[M − H − C_9_H_16_O]^−^	9-Hydroperoxy-octadecadienoic acid
68	27.37	309.2086	309.2071	4.6	C_18_H_30_O_4_	211.1333[M − H − C_6_H_12_O]^−^, 197.1180[M − H − C_7_H_11_ − H_2_O]^−^	11-Hydroperoxy octadecatrienoic acid
69	28.35	311.2241	311.2228	4.1	C_18_H_32_O_4_	293.2138[M − H − H_2_O]^−^, 185.1181[M − H − C_8_H_14_O]^−^, 171.1030[M − H − C_9_H_16_O]^−^	9-Hydroperoxy-octadecadienoic acid
70	28.89	329.2234	329.2333	0.3	C_18_H_34_O_5_	211.1351[M − H − C_6_H_14_O_2_]^−^, 171.1025[M − H − C_8_H_14_O_3_]^−^	Trihydroxy octadecenoic acid
71	29.27	311.2239	311.2228	3.6	C_18_H_32_O_4_	293.2133[M − H − H_2_O]^−^, 185.1183[M − H − C_8_H_14_O]^−^, 171.1029[M − H − C_9_H_16_O]^−^	9-Hydroperoxy-octadecadienoic acid
72	30.29	291.1980	291.1966	5.0	C_18_H_28_O_3_	273.1857[M − H − H_2_O]^−^, 247.2078[M − H − H_2_O − CO_2_]^−^	12-Oxo-phytodienoic acid
73	30.56	559.3142	559.3124	3.3	C_28_H_48_O_11_	277.2186[M − H − C_10_H_18_O_9_]^−^	Dirhamosyl linolenic acid
74	30.85	293.2135	293.2122	4.3	C_18_H_30_O_3_	275.2031[M − H − H_2_O]^−^, 183.1390[M − H − C_7_H_10_O]^−^, 171.1032[M − H − C_9_H_14_]^−^,	Hydroxy octadecatrienoic acid
75	31.29	293.2135	293.2122	4.3	C_18_H_30_O_3_	275.2016[M − H − H_2_O]^−^, 223.1335[M − H − C_5_H_10_]^−^, 195.1387[M − H − C_6_H_10_O]^−^	Hydroxy octadecatrienoic acid
76	32.80	291.1977	291.1966	4.0	C_18_H_28_O_3_	211.1334[M − H − C_6_H_8_]^−^, 197.1183[M − H − C_7_H_10_]^−^, 185.1177[M − H − C_8_H_10_]^−^,	Oxo-octadecatrienoic acid
77	33.62	295.2283	295.2279	1.5	C_18_H_32_O_3_	277.2158[M − H − H_2_O]^−^, 195.1387[M − H − C_6_H_12_O]^−^, 171.1026[M − H − C_9_H_16_]^−^	9-Hydroxy-10, 12-octadecadienoic acid Hydroxy octadecadienoic acid
78	34.81	293.2135	293.2122	4.3	C_18_H_30_O_3_	249.2215[M − H − CO_2_]^−^, 195.1385[M − H − C_6_H_10_O]^−^, 179.1071[M − H − C_6_H_10_O_2_]^−^, 113.0965[M − H − C_11_H_16_O_2_]^−^	Oxo-octadecadienoic acid
79	35.48	293.3133	293.2122	3.6	C_18_H_30_O_3_	185.1179[M − H − C_8_H_12_]^−^, 125.0961[M − H − C_9_H_12_O_3_]^−^	Oxo-octadecadienoic acid
80	36.00	293.2123	293.2122	0.2	C_18_H_30_O_3_	185.1157[M − H − C_8_H_12_]^−^, 125.0963[M − H − C_9_H_12_O_3_]^−^	Oxo-octadecadienoic acid
Others							
1	3.06	341.1101	341.1089	3.4	C_12_H_22_O_11_	179.0595[M − H − C_6_H_10_O_5_]^−^, 161.0470[M − H − C_6_H_12_O_6_]^−^, 113.0229[M − H − C_7_H_16_O_8_]^−^	Sucrose
2	3.06	179.0566	179.0561	2.6	C_6_H_12_O_6_	113.0234[M − 2H_2_O − CH_2_OH]^−^,	Monose
4	5.40	305.1598	305.1606	−2.6	C_14_H_26_O_7_	175.0250, 161.0230, 133.0296	Unidentified
6	5.80	137.0247	137.0244	2.0	C_7_H_6_O_3_	-	Protocatechualdehyde
7	5.80	299.0783	299.0772	3.5	C_13_H_16_O_7_	137.0270	Unidentified
8	6.31	305.1616	305.1606	3.4	C_14_H_26_O_7_	289.1306, 272.1043, 247.1083, 148.0521, 134.0375	Unidentified
12	8.02	391.0828	391.0823	1.3	C_22_H_16_O_7_	193.0513, 178.0272, 149.0605, 134.0374	Unidentified
29	10.85	177.0204	177.0201	1.8	C_9_H_6_O_4_	177.0180[M − H]^−^, 149.0234[M − H − CO]^−^, 133.0285[M − H − CO_2_]^−^, 105.0336[M − H − C_2_O_3_]^−^	Dihydroxycoumarin
31	11.12	431.1938	431.1935	0.6	C_20_H_32_O_10_	385.1837, 223.1382, 205.1203, 163.1131, 119.0333, 113.0281, 101.0234	Hydroxy-2,4,4-trimethyl-3-(3-oxobutyl)-2-cyclohexen-1-one glucoside
39	12.46	723.5092	723.5089	0.4	C_41_H_72_O_10_	677.5014, 659.4905, 550.4370, 451.3300, 433.316, 367.2732, 341.2932, 309.2213, 225.1609, 207.1497, 143.0814, 125.0709,	Unidentified
52	16.62	193.0513	193.0508	2.4	C_10_H_10_O_4_	161.0244, 133.0296	Unidentified
53	17.18	201.1145	201.1144	0.2	C_10_H_18_O_4_	183.1026[M − H − H_2_O]^−^, 139.1128[M − H − H_2_O − CO_2_]^−^	Dibutyl oxalate
61	23.00	307.1928	307.1915	4.4	C_18_H_28_O_4_	235.1346[M − H − C_5_H_12_]^−^, 211.1343, 185.1188, 137.0966	Dihydrocapsiate
62	24.18	311.1878	311.1878	−0.2	C_17_H_28_O_5_	293.1750[M − H − H_2_O]^−^, 267.1966[M − H − CO_2_]^−^	Dihydroartemisinin ethyl ether
63	25.37	305.1770	305.1758	4.0	C_18_H_26_O_4_	249.1499, 135.0809	Unidentified

**Table 4:** Independent variables and their levels used for Box—Behnken design.

Factors	Coded Symbols	Levels		
		− 1	0	1
Liquid-to-solid ratio (mL/g)	X_1_	30	40	50
Power (W)	X_2_	40	60	80
Time (min)	X_3_	300	350	400

## Supplementary Materials

The following are available online, Table S1: Correlation analysis between bioactive compounds and *α*-glucosidase inhibitory activity in methanol extracts of CDL.

## Abbreviations

CDL	*Ceratophyllum demersum L*
AGIs	α-glucosidase inhibitors
RSM	response surface methodology
UAE	ultrasonic-assisted extraction
BBD	Box-Behnken design
TFC	total flavonoid content
TPC	total phenolic content
pNPG	*p*-nitrophenyl-α-d-glucopyranoside
BPC	base peak chromatogram
ESI	electrospray ionization source
ANOVA	one-way analysis of variance
