# Supplementary material for: Ultrasound-Assisted Extraction Optimization of α-Glucosidase Inhibitors from Ceratophyllum demersum L. and Identification of Phytochemical Profiling by HPLC-QTOF-MS/MS
Source: Molecules. 2020 Oct 1;25(19):4507. doi: 10.3390/molecules25194507 (PMC7582508; doi:10.3390/molecules25194507)
Supplement: Supplementary file 1 [file molecules-25-04507-s001.pdf]

Supplementary material

Table 1. Correlation analysis between the bioactive compounds and *α*-glucosidase inhibitory activity of CDL extracts.

	TPC	TFC	*α*-Glu
TPC	1	−0.204	−0.449
TFC		1	−0.648 **
α-Glu			1

Note: TPC: Total phenolic content; TFC: Total flavonoid content; *α*-Glu: *α*-glucosidase inhibitory activity; ** represents extreme correlation ($P < 0.01$); * represents significant correlation ($P < 0.05$).
